# Idiopathic granulomatous mastitis: challenges of treatment in iranian women

**DOI:** 10.1186/s12893-021-01210-6

**Published:** 2021-04-21

**Authors:** Leyla Shojaee, Nasrin Rahmani, Siavash Moradi, Asieh Motamedi, Gholamali Godazandeh

**Affiliations:** 1 Department of Surgery, School of Medicine, Mazandaran University of Medical Sciences, Sari, Iran; 2 Community Medicine Specialist, Gastrointestinal Cancer Research Center, Mazandaran University of Medical Sciences, Sari, Iran; 3 Mazandaran University of Medical Science, Sari, Iran

**Keywords:** Idiopathic granulomatous mastitis, Recurrent, Treatment

## Abstract

**Background and objective:**

As a chronic inflammatory disease of an unknown origin, the treatment of granulomatous mastitis has always been controversial. According to some researchers, surgical treatment and certain medications, especially steroids, are more effective in treating the disease. This study aimed at evaluating the results of treatment in a group of patients with granulomatous mastitis.

**Materials and methods:**

This longitudinal cohort study evaluated the treatment outcomes of 87 patients with pathology-confirmed granulomatous mastitis referred to the surgical clinic of Central Hospital in Sari, Iran. Demographic, clinical, and pathological information, treatment methods and results, and the recurrence rate were analyzed.

**Findings:**

A total of 87 female patients with granulomatous mastitis aged 22–52 years with a mean age of 34 years were evaluated. All patients had palpable masses; the breast masses were painful in 48.3% of patients, and 55.2% of patients suffered from erythema and inflammation, and8% had fistulas and ulcers at the inflammation site. The patients were followed-up for an average duration of 26 months (8–48 months) after treatment and recovery. The overall recurrence rate was 24.1%, and the recurrence rate was 29.4% in patients underwent surgery, 34.8% in patients received high-dose prednisolone, and 17% in those received low-dose prednisolone together with drainage (*p* < 0.001).

**Conclusions:**

According to the results, the low-dose prednisolone plus drainage was more effective with a lower recurrence rate than only surgical excision or high-dose prednisolone. In fact, the use of minimally invasive methods such as drainage plus low-dose steroids is a more effective method with fewer side effects than the other two methods.

## Introduction

Idiopathic granulomatous mastitis (IGM) is a chronic benign inflammatory disease of the breast that often affects women of reproductive age [1]. Several factors have been identified as IGM risk factors, including reaction to chemicals such as oral contraceptive pills (OCP), infectious diseases, and autoimmune diseases in response to immunological stimuli due to milk flow in epithelial lobules [2, 3].

However, the etiology of IGM is still unknown, raising debates on its treatment and resulting in no single cure or treatment strategy. Various medical treatments have been reported for IGM including topical and systemic corticosteroids and antibiotics which decreased the recurrence rate [4], as well as methotrexate [5], azathioprine [6], and colchicine. In fact, corticosteroids and surgical excision are known as current treatments [2, 7–10]. Surgical treatment of IGM includes local abscess drainage and wide excision or mastectomy. Methotrexate treatment is usually not common in the treatment of patients with idiopathic granulomatous mastitis and is mostly used in cases resistant to common therapies with side effects of corticosteroids and has been associated with good results in this group of patients [11].

There are reports on the good results obtained from surgical treatment plus steroids consumption [12]. The disease may be locally invasive with a risk of recurrence. Recurrence rates of 5 to 50% have been observed following various treatment methods [13–15]; for instance, a recurrence rate up to 50% has been reported after surgical excision [16]. Recognizing the effective factors of IGM recurrence is as important as its causes. Breastfeeding, pregnancy, breast infection, and smoking are the well-known recurrence factors [17]. The type of treatment during remission is also known to be an effective factor. This study aims at investigating the outcomes of treatment in three groups of patients with IGM.

## Materials and methods

The information of 87 patients with histopathological-confirmed IGM visiting the surgery clinic of Central Hospital in Sari, Iran, from June 2015 to January 2020 was recorded. Idiopathic granulomatous mastitis (IGM) is a rare disease and only 113 patients had referred to our Central Teaching Hospital during our study period (2015–2020). Patients’ diagnosis was performed based on imaging and then biopsy of inflammatory mass and pathology confirmation, 26 patients (17 patients were in the high-dose corticosteroids and 9 patients were in the low-dose corticosteroids) were excluded from the study. In patients receiving high-dose corticosteroids, one patient as a result of venous thromboembolism and fifteen patients in the early stages of treatment as a result of weight gain and gastrointestinal problems and hyperglycemia withheld from continuing treatment with corticosteroids and one patient did not refer and could not be followed up. Those patients who receiving low-dose corticosteroids by drainage, 5 patients withheld to continue receive corticosteroids due to weight gain and 4 patients due to hyperglycemia. All patients who withheld receive corticosteroids and patients who recurred after recovery, as well as patients who did not respond to treatment and were resistant to corticosteroids, were treated with methotrexate which their results were not reported and in the next article, results of methotrexate treatment will be discussed. Patients with weaker diseases and the lesion were limited to a quadrant of the breast and surgical resection was performed with a suitable margin so that lumpectomy with a healthy margin was performed without significant deformity.

All patients underwent clinical examination and imaging, including ultrasound in all patients and mammography in patients over 40 years. The definitive diagnosis of the disease was made through core biopsy or excisional biopsy. Patients were divided into three treatment groups, namely surgical excision (Group 1), high-dose prednisolone (Group 2), and low-dose prednisolone plus drainage (Group 3). Group 1 consisted of 17 patients with granulomatous mastitis underwent surgical excision of the breast inflammatory tissue with healthy marginal in the operating room under general anesthesia. Group 2 was treated with high-dose prednisolone (50 mg) twice daily for two weeks which was tapered to 50 mg, 25 mg, 10 mg, and 5 mg daily every two weeks based on response to treatment and clinical examination. Patients in Group 3 first underwent a core needle (G 16) biopsy after local anesthesia with 1% lidocaine in the inflammatory mass palpation site. In addition, 8 to 10 cuts of the inflammation site along with the skin (up to ​​5 mm^2^) were biopsied and sent for pathological examination. After repetition of inflammatory tissue peeling, a 4 cm long, 3 mm diameter plastic tube was fixed at the biopsy site for drainage and bandaged. At the same time, patients in Group 3 received steroids 30 mg/day for 2 weeks which was tapered to 20 mg/day for 4 weeks, 10 mg/day for 2 weeks, and then 5 mg/day for 2 weeks.

The prescribed corticosteroids’ dose was calculated on the basis of 1 mg/kg. Accordingly, those patients who received a dose of more than one mg/kg would be classified in the high dose group and those patients who received a dose lower than the weight would be classified in the low-dose group. High-dose corticosteroids are started with 100 mg and gradually taper and low-dose corticosteroids are started with 30 mg, which is fully described in the present article. Those patients who receive low-dose corticosteroids with peeling drainage As Deng et al. [18] attempted to remove inflammatory tissue in the affected area of ​​the breast to reach a healthy margin of breast tissue by vacuum, the purpose of our study is to remove more tissue from the biopsy area by using a core needle No. 16 to create a path for drainage of secretions and reduction of pressure in the inflamed area due to the accumulation of inflammatory fluid and placing the drain for a week until the oral corticosteroid reaches the appropriate level to control inflammation. The purpose of peeling in present study was not to reach a healthy margin and to create a deliberate fistula for better drainage to prevent pressure on the surrounding tissues and to create multiple spontaneous fistulas in the breast.

The biopsy site drain was removed after a week. All pathology slides were stained with hematoxylin-eosin, and with special stains such as Gram and Ziehl-Nielsen for tuberculosis and periodic acid-Schiff for fungal infection. After recovery and discontinuation of treatment, the patients were examined every two to three months and then underwent clinical and radiological examination (ultrasound) every 6 months in terms of recurrence symptoms including edema, erythema, inflammatory mass, fistula, and secretions. Regarding ethical considerations, the disease type, the treatment method, and the disease complications and their treatment were fully explained to all patients. All methods were carried out in accordance with relevant guidelines and regulations.

### Data analysis

The Kolmogorov-Smirnov statistical test was used to examine the data distribution. The quantitative data were then described using the mean (standard deviation) and median (interquartile range), and the qualitative data were described using the frequency (percentage). It is worth noting that all descriptions and analyses were performed in IBM SPSS21, and a two-way *p*-value of less than 0.05 was considered significant in all cases.

## Results

A total of 87 patients with IGM were evaluated and treated from 2015 to 2020 and then followed-up averagely for 26 months (8–48 months).

According to the Table 1, 9.2% of patients are under 25 years old, 72.4% of patients are between 25 and 40 years old and 18.4% are over 40 years old. The results of one-way chi-square test showed that the age distribution of patients was not the same at the error level of 0.05, so that the highest frequency is related to the age group of 25–40 years. Also, the mean age of participants was 34.11 ± 7.23 years. Recurrence patients and patients without recurrence did not have a statistically significant difference in age (P = 0.949).

None of the patients were menopause. None of the patients had a history of smoking, 44.9% had a history of contraceptive consumption, and 73.6% had a history of breastfeeding. The serum prolactin levels were normal in 35.6% and high in 64.4% of the patients. All patients had a palpable mass, but the breast mass was painful in 48.1% of patients; 55.2% suffered from erythema and skin edema, and 8% of patients had ulcer and fistula in the breast (Table 2).

Side effects for those patients who have completed treatment on high dose corticosteroids included:

All patients gained weight (3–13 kg). Hyperglycemia has been observed in 7 patients, headache in 8 patients, gastrointestinal problems but not digestive bleeding in 12 patients; however, there was mild to severe stomachache due to gastritis, although they were treated with pantoprazole. Hair loss was seen in 6 patients. Mild blurred vision in 2 patients who were referred to an ophthalmologist for cataract examination.

Those patients who receive low-dose corticosteroids with drainage had fewer side effects including weight gain 2 to 5 kg and had mild hypoglycemia (upper limit) in 4 patients and gastrointestinal problems in the form of stomachache in 5 patients. No hair loss and gastrointestinal bleeding was observed. Three patients also had Headache.

**Table 1:** Age distribution of patients with GM

Age Group (years)	Total prevalence of patients (%)	Patients with recurrence (%)	Patient without recurrence (%)	P-value
< 25	8 (9.2%)	2 (8%)	6 (9.7%)	0.949
25–40	63 (72.4%)	18 (72%)	45 (72.6%)	
40<	16 (18.4%)	5 (20%)	11 (17.7%)	
Total	87	25	62	

**Table 2:** Characteristics of patients included into the study

Palpable mass	100%
+Painful mass	42 (48.3%)
+Erythematous mass	55.2%
+Ulcer and fistula	8%
Unilateral right breast	54 (62.2%)
Unilateral left breast	30 (34.4%)
Bilateral	3 (3.4%)
One quadrant	17 (19.5%)
> One quadrant	70 (80.5%)
Cigarette usage	
Yes	0
No	87 (100%)
OCP (Oral Contraceptive)	
Yes	13 (14.9%)
No	74 (85.1%)
Breast feeding	
Yes	64 (73.6%)
No	23 (26.5%)
BMI (Body Mass Index)	
≤ 30	39(44.8%)
> 30	48(55.2%)
Serum prolactin level	
Normal	31 (35.6%)
High	56 (64.4%)

IGM was confined to one breast quadrant in 19.5% of patients and involved multiple quadrants in 80.5% of patients, 100% underwent ultrasound, and 30.1% underwent both ultrasound and mammography.

Regarding treatment, 96% of patients first received antibiotics, 26.1% received high-dose prednisolone of which 34.8% recurred during the follow-up period; 51.1% received low-dose prednisolone plus drainage of which 17% experienced recurrence; 34.8% received high-dose prednisolone experienced recurrence and patients underwent surgical excision of which 29.4% recurred. There was not a statistically significant difference between the three groups in terms of recurrence, but the lowest recurrence rate was observed in the low-dose corticosteroid plus drainage group, p-value < 0/001) (Table 3).

The results in Table 3 showed that among all patients, 26.4% received high dose of drug, 54% received low dose and 19.5% underwent surgery. Among those who received high dose of drug, 39.1% had recurrence of the disease. The ratio of recurrence and non-recurrence in this group receiving high dose was statistically similar (p = 0.202), but among those who received low dose of the drug, it was observed that 19.1% had recurrence, which was statistically in this group, the ratio of patients without recurrence of the disease was significantly low (p < 0.001). In the group receiving surgical treatment, 41.2% of patients in this group of treatment had recurrence of the disease (P = 0.315). In total, 28.7% recurrence was seen in all patients, regardless of the type of treatment.

**Table 3:** Treatments and recurrence

Intervention group	Frequency in total patients (%)	Patients without recurrence	Patients with recurrence	Percent of recurrence in each group	P-value^a^	P-value^c^
High-dose corticosteroid	23 (26.4%)	14 (22.6%)	9 (36.0%)	39.1%	0.202	0.100
Low-dose corticosteroid plus drainage	47 (54.0%)	38 (61.3%)	9 (36.0%)	19.1%	< 0.001	
Surgical excision	17 (19.5%)	10 (16.1%)	7 (28.0%)	41.2%	0.315	
Total number	87 (100%)	62 (100%)	25 (100%)	28.7%	< 0.001	
P-value^b^	< 0.001	< 0.001	0.825	0.098		

^a^Binominal Test

^b,c^Chi-Square

Although the correlation was not significant using chi-square test in the agreement table (p = 0.100), but in a more detailed study, it was observed that with a recurrence ratio of 0.5 according to the sample size of each treatment group, the recurrence rate in the group low-dose therapy was lower than other treatment groups. Comparison of the recurrence ratio between the three treatments in pairs shows that the high-dose and surgical treatment groups were not different (p = 0.448), but these two groups were significantly different from the low-dose treatment group, so that test a sequence for two-sentence ratio comparison between the two groups showed that the recurrence rate of the disease at low dose was lower than the recurrence rate at high dose (p = 0.044) and also the recurrence rate at low dose was lower than the recurrence rate at surgical treatment (p = 0.048). The recurrence ratio in the surgical and high dose groups was almost twice the recurrence ratio in the low dose group.

## Discussion

Idiopathic granulomatous mastitis (IGM) is a disease of unknown origin and is actually a chronic inflammatory disease that mainly affects women of reproductive age [19–21], because its appearance can mimic the symptoms of breast cancer. As a result, the diagnosis must be confirmed histologically before starting the treatment [22, 23]. The disease etiology is unknown. However, it has been proposed that IGM may result from damage to the duct epithelium due to chemical or infectious stimuli which may lead to fat extravasation and ultimately granulomatous response and migration of macrophages and lymphocytes to the breast tissue [24–26] The unknown cause of IGM has led to treatment challenges among researchers in various studies.

In one study, high-dose prednisolone was recognized as the selective treatment which was associated with a low recurrence rate during follow-up of patients. In this study, 36%, 8%, and 56% of patients received only medical treatment, only surgical excision, and a combination of surgical and medical treatment, respectively. Among those treated medically, 39% received oral steroids as the first choice of medical treatment, 37% received antibiotics, 13% received antibiotics plus steroids, and 1% received methotrexate; the overall recurrence rate was 17% [27]. In a study examining the results of treatment in 200 patients with granulomatous mastitis, high-dose corticosteroids were associated with better remission and fewer recurrences [28].

In another study, 93% of patients underwent a surgical procedure. Out of the 43 patients with a median age of 34 years, 18 underwent surgical resection, 20 underwent surgical excision plus drainage, and only 3 received prednisolone post surgically. The patients were followed-up for averagely 15 months, and a recurrence rate of 23% was obtained. In this study, surgical excision was introduced as the best treatment, which was both diagnostic and therapeutic with wide excision [29]; this method has also been recommended in other studies [30, 31]. In contrast to this study, in another study comparing the results of surgical treatment with prednisolone, recurrence was higher in the surgical group than in steroid therapy [32].

The use of corticosteroids is still controversial. Some authors recommend steroid consumption after confirmation of the diagnosis [30, 33]. Similar to granulomatous thyroiditis and psoriasis, granulomatous mastitis has shown a good response to steroid therapy, therefore, IGM seems to have an autoimmune basis [24].

In this study, different recurrence rates were found for the three groups of patients who received different treatments (*p* < 0.001). Despite the initial response and faster recovery, the recurrence rate of patients who underwent only surgical excision was 29.4%. IGM was recurred in the non-involved quadrant in all 5 patients who recurred. It should be noted that the remission period was 8–38 months with a mean period of 25 months.

The highest recurrence rate was found for patients who received high-dose corticosteroid; 34.8% of these patients relapsed faster than other groups after discontinuation of the treatment. Most relapses were in the primary quadrant (90%). In contrast, the lowest recurrence rate of 17% was found in the group received low-dose corticosteroid plus drainage. These patients had fewer side effects without any deformity and surgical scar. Moreover, among other patients with recurrence, the remission time was longer with an average duration of 30 months (14–44 months) (p < 0.001).

Surgical excision along with steroids has been recommended by some authors, and corticosteroids have been limited in some studies. The recurrence rate after excision alone has been reported to be 16–50% [9, 34].

The unknown cause of IGM has led to treatment challenges among researchers in various studies. Actually, IGM can have different presentation and severity in different patients. by creating these different manifestations, many differential diagnoses are made, from breast cancer to tuberculosis and sarcoidosis and fungal diseases to various autoimmune diseases such as rheumatoid arthritis, granulomatous diseases, etc. For this reason, it can be diagnosed by rejecting other diseases using serological tests and culture and biopsy of the breast. In all autoimmune diseases, the mammary glands can be one of the targets. Organs that are specifically targeted by the disease, such as autoimmune thyroiditis, autoimmune granulomatosis (sarcoidosis) and etc. Therefore, diagnosis of autoimmunity is necessary for timely treatment and initiation of corticosteroids [35–37].

In a study, steroid was administered after vacuum-assisted biopsy by mammotom resulted in a success rate of 81.5%; in this study, the overall recurrence rate was 18.5%, the mean follow-up was 12 months, and the patients received 30 mg/day prednisolone for two weeks after which the drug was tapered to 20 mg/day, 10 mg/day, and 5 mg/day each for two weeks [18].

In our study, a combination of minimally invasive surgery, as drainage or deliberate creation of a fistula to drain discharge and reduce inflammation, along with low-dose prednisolone for 10 weeks resulted in a better treatment response and a lower recurrence rate. Surgical excision alone cannot be performed in all patients due to the extent of the lesion, and excision with healthy margins results in breast defects and deformity. Although surgical excision was performed in patients with only one quadrant involvement and the disease was not extensive, the recurrence rate was higher in the surgical excision than the low-dose steroid plus drainage group, and all cases of recurrence occurred in the non-involved quadrant indicating the autoimmune nature of the disease. It seems that a combination of minimally invasive therapies in which drainage is established and therapeutic beneficial effects of low-dose corticosteroid are used to suppress the immune system can result in fewer side effects and more effective treatment.

## Conclusions

As an autoimmune disease, idiopathic granulomatous mastitis appears to have different manifestations and severity in different patients; therefore, a same treatment cannot be used for all patients with histologically-diagnosed IGM. The disease is self-limited and remitted spontaneously in some patients. However, most patients require treatments such as corticosteroids or surgical excision. Due to the severity and extent of the disease in some patients, surgical excision to achieve a healthy margin is not possible without severe breast deformity; therefore, surgical treatment can be limited to patients with limited involvement, those with corticosteroid side effects, or those resistant to other treatments. However, in contrast with extensive surgeries, minimally invasive surgeries performed as a surgical procedure, a temporary drain for discharge, and low-dose steroids for 10 weeks are associated with minimal recurrence, fewer high-dose corticosteroid-induced side effects, and minimal scare and deformity.

## Data Availability

The datasets used and/or analyzed during the current study available from the corresponding author on reasonable request.
